# Inflammatory Markers and Saphenous Vein Graft Stenosis: Insights into the Use of Glucose-to-Lymphocyte Ratio as a Prognostic Marker

**DOI:** 10.3390/jcm14082634

**Published:** 2025-04-11

**Authors:** Aydin Tuncay, Yucel Yilmaz, Oguzhan Baran, Saban Kelesoglu

**Affiliations:** 1 Department of Cardiovascular Surgery, Faculty of Medicine, Erciyes University, 38280 Kayseri, Türkiye; atuncay@erciyes.edu.tr; 2 Department of Cardiology, University of Health Sciences, Kayseri City Training and Research Hospital, 38060 Kayseri, Türkiye; dryyilmaz@hotmail.com (Y.Y.); oguzhanbaran2009@hotmail.com (O.B.); 3 Department of Cardiology, Faculty of Medicine, Erciyes University, 38280 Kayseri, Türkiye; 4 Kosk Mah. Prof. Dr. Turhan Feyzioglu Cad. Erciyes Universitesi Saglik Uygulama ve Arastirma Merkezi No: 42, Faculty of Medicine, Heart Hospital, Erciyes University, 38039 Kayseri, Türkiye

**Keywords:** coronary artery bypass graft, saphenous vein graft stenosis, glucose-to-lymphocyte ratio, inflammation

## Abstract

**Background:** Coronary artery bypass grafting (CABG) for the treatment of ischemic heart disease is still considered an effective treatment option to improve clinical outcomes and reduce mortality. However, the patency rates of saphenous vein grafts (SVGs) are significantly lower compared to those of arterial grafts. Atherosclerosis has emerged as one of the main causes of SVG stenosis (SVGS), especially stenoses that develop after one year. In this study, we aimed to investigate the association of glucose-to-lymphocyte ratio (GLR), a novel inflammatory biomarker, with LVG patency status in patients undergoing CABG surgery. **Methods:** A total of 778 patients who were diagnosed with chronic coronary syndromes (CCS) according to the 2019 ESC guidelines for the diagnosis and treatment of CCS; had undergone CABG more than one year previously; and had at least one SVG used during surgery were included in this study. GLR was calculated as blood glucose level (mg/dL) divided by lymphocyte count (K/uL). **Results:** SVGS was detected in 341 patients, while SVGs were intact in 437 patients. Patients with SVGS had a higher prevalence of diabetes mellitus (DM) (*p* = 0.002) and significantly higher blood glucose levels (*p* < 0.001). In addition, the interval between CABG operation and coronary angiography (CAG) was longer in the SVGS group (*p* < 0.001). Neutrophil levels were higher, and lymphocyte levels were lower in this group (*p* = 0.010 and *p* = 0.034, respectively). Neutrophil/lymphocyte ratio (NLR), platelet/lymphocyte ratio (PLR), glucose/lymphocyte ratio (GLR) and high-sensitivity C-reactive protein (CRP) levels were significantly higher in patients with SVGS (*p* < 0.001 for all). According to multivariate logistic regression analysis, DM, CRP level, time since CABG, and GLR were identified as independent predictors of SVGS (*p* = 0.004, *p* = 0.048, *p* < 0.001, and *p* < 0.001, respectively). ROC analysis showed that SVGS could be predicted with 75.8% sensitivity and 68.6% specificity when the cut-off value for GLR was >315.5 (area under the curve [AUC]: 0.801, 95% CI: 0.765–0.837, *p* < 0.001). **Conclusions:** Higher GLR levels are associated with SVGS in patients with coronary artery disease.

## 1. Introduction

Ischemic heart diseases are among the leading causes of mortality and morbidity worldwide, and coronary artery bypass grafting (CABG) remains an effective option for the treatment of these diseases to reduce adverse clinical outcomes. CABG is one of the most commonly performed cardiac surgical procedures with more than half a million procedures performed worldwide each year [1].

Left internal mammarian artery (LIMA) and saphenous vein grafts (SVG) continue to be the standard grafting materials for CABG [2]. The use of SVGs as a surgical conduit in CABG remains widespread, despite the literature clearly demonstrating that SVGs have low long-term patency rates [3,4,5,6,7]. In recent years, advances in the diagnosis, treatment, and follow-up of patients with coronary artery disease (CAD) have led to significant improvements in prognosis after CABG. However, due to the increasing need for revascularization following CABG, the diagnosis and treatment of SVG stenosis (SVGS) is becoming an increasingly important issue in cardiology and cardiac surgery practice [8,9,10].

Neointimal hyperplasia is the main cause of SVGS between 1 and 12 months, whereas atherosclerotic processes are the main cause of narrowing after 1 year [11,12]. Inflammation plays a critical role in the pathogenesis of atherosclerotic heart disease. In the literature, studies have demonstrated the importance of inflammation markers such as neutrophil-to-lymphocyte ratio (NLR), platelet-to-lymphocyte ratio (PLR), and C-reactive protein (CRP) in determining the severity and prognosis of CAD. Similarly, studies have been published supporting that hematologic indices such as NLR, lymphocyte-to-monocyte ratio (LMR), and systemic immune-inflammation index (SII) may be important markers for predicting mortality and SVG patency after CABG [13,14,15,16].

However, there is no study in the literature examining the relationship between glucose-to-lymphocyte ratio (GLR), a recently described new inflammation parameter, and atherosclerotic disease in patients undergoing CABG. In this study, we aimed to investigate whether there is an association between GLR and SVGS in patients undergoing CABG.

## 2. Material and Methods

### 2.1. Patient Selection

This study included 778 CABG patients who underwent coronary angiography (CAG) after noninvasive stress tests (positive stress test results and/or ischemia findings on myocardial perfusion scintigraphy) performed in the cardiology department of our hospital between 2016 and 2024, with typical clinical symptoms of chest pain and/or suspected myocardial ischemia.

These patients were diagnosed according to the 2019 European Society of Cardiology (ESC) Chronic Coronary Syndromes (CCS) diagnosis and treatment guidelines, had undergone CABG surgery more than one year previously, and had at least one SVG used during the operation [17]. SVGS was defined as narrowing of the SVG by 50% or more.

### 2.2. Exclusion Criteria

Exclusion criteria were a history of acute coronary syndromes in the last three months, LIMA disease, decompensated heart failure, severe valvular heart disease, hematologic disease, malignancy, severe lung disease, severe renal impairment (estimated glomerular filtration rate [eGFR] < 30 mL/min/1.73 m^2^) or liver failure, infection, or chronic inflammatory and/or autoimmune diseases.

### 2.3. Ethical Consent

The study was conducted in accordance with the guidelines outlined in the Declaration of Helsinki. The Institute of Research Ethics of our hospital approved the non-experimental design of this retrospective study. In addition, a waiver of informed consent was approved by the ethics committee due to its retrospective design based on patient records. (Ethical approval; 28.11.2023/955).

### 2.4. Definitions

Diabetes mellitus (DM) was defined as postprandial blood glucose > 200 mg/dL and/or fasting blood glucose > 126 mg/dL and/or the use of anti-diabetic medication. Systolic blood pressure/diastolic blood pressure of at least 140 mm Hg/90 mm Hg or the use of anti-hypertensive medication was considered hypertension (HT). Total cholesterol levels above 200 mg/dL and/or the use of anti-hyperlipidemic drugs were considered hyperlipidemia. Patients who smoked for at least 6 months/last year were defined as smokers [18].

#### Coronary Angiography and Percutaneous Coronary Intervention

All patients underwent coronary angiography (CAG) within 24 h of hospitalization. CAG was performed through a radial or femoral artery approach, depending on physician preference, and coronary arteries, main branches, and conduit vessels were evaluated. Judkins catheters were routinely used to cannulate the left and right coronary ostia; additional catheters were used as needed. Imaging of the ascending aorta was performed with a pigtail catheter when necessary to assess SVG. CAG results were evaluated by at least two experienced invasive cardiologists. Invasive cardiologists recommended appropriate treatment strategies to patients based on angiography findings, according to current treatment guidelines [17].

All patients were treated with percutaneous coronary intervention (PCI) in the same session as CAG when necessary. Prior to CAG, patients received adjuvant antiplatelet therapy with aspirin (300 mg), clopidogrel (300–600 mg), or ticagrelor (180 mg). Unfractionated heparin was given according to body weight up to 100 IU/kg before PCI and tirofiban was added as needed. After the procedure, routine antiplatelet therapy was initiated for patients [17].

### 2.5. Laboratory Analysis

Blood samples were collected from all patients the next morning following admission to the hospital. Antecubital venous blood samples were collected. Venous blood samples were collected for comprehensive metabolic panels and complete blood count. Biochemical tests were performed on an automatic analyzer (COBAS^®^ c701, Roche Diagnostics, Mannheim, Germany). Hematologic parameters were stored at 4 °C and evaluated using an autoanalyzer (Sysmex K-1000 Hematology Analyzer, Kobe, Japan) as soon as possible after sampling (maximum within 30 min). NLR was calculated as the number of neutrophils divided by the number of lymphocytes. PLR was calculated as platelet count divided by lymphocyte count. GLR was calculated as blood glucose level (mg/dL) divided by lymphocyte count (K/uL).

#### Transthoracic Echocardiography

Each participant underwent transthoracic echocardiography. All measurements were performed using a machine equipped with a 3.5 MHz transducer (Vivid 5, GE Medical System, Horten, Norway). The Teicholz and/or Simpson method was used to assess left ventricular ejection fraction (LVEF).

### 2.6. Statistical Analyses

Statistical analyses were performed using SPSS 21.0 for Windows (SPSS Inc., Chicago, IL, USA). The distribution of quantitative variables was checked with the Shapiro–Wilk test. Descriptive data were presented as mean ± standard deviation and median (interquartile range, IQR) depending on the normality of the distribution. Median and quartile ranges were given for non-normally distributed variables. Independent samples *t*-test was used to compare quantitative variables with normal distribution, and Mann–Whitney U test was used for quantitative variables without normal distribution. Categorical variables were compared using the chi-square test. The effects of different variables on the development of SVGS were calculated using univariate analysis. For multivariate regression analysis, parameters with a *p* value < 0.10 in univariate analysis were included in the model. However, to avoid multicollinearity, parameters that interacted with each other (e.g., platelet count, SII, NLR, and PIV) were not entered into the model and therefore multivariate regression analysis was performed separately with inflammatory parameters. Cut-off levels for PIV, SII, and CAR in predicting disease occurrence were determined by receiver operating characteristic (ROC) curve analysis. *p* values below 0.05 were accepted to indicate statistical significance.

## 3. Results

Coronary angiography was performed in all 778 CABG patients included in this study. Of these patients, 341 had SVGS and 437 had intact SVGs. There was no significant difference in age, gender, body mass index (BMI), and LVEF between the two groups (*p* > 0.05).

In terms of risk factors, both groups showed similar characteristics in terms of HT, hyperlipidemia, and smoking (*p* > 0.05). However, the rate of DM was statistically higher in patients with SVGS (88 (20.1%) vs. 102 (29.9%), *p* = 0.002). No statistical difference was observed between the groups in terms of medications used (aspirin, beta-blockers, angiotensin-aldosterone antagonists, statins, clopidogrel, nitrates) (*p* > 0.05) (Table 1).

The laboratory results of patients with SVGS and intact SVGs are summarized in Table 2. Creatinine, AST, ALT, total cholesterol, high-density lipoprotein cholesterol (HDL-C), low-density lipoprotein cholesterol (LDL-C), and triglyceride levels were not significantly different between the two groups (*p* > 0.05). However, blood glucose levels were statistically higher in the SVGS group (89 (78–106) vs. 113 (88–160), *p* < 0.001).

In terms of hematologic parameters, no significant difference was found in hemoglobin, platelet, and white blood cell (WBC) levels between both groups (*p* > 0.05). Neutrophil count was higher in the SVGS group (3.9 (2–7.4) vs. 4.2 (3–6.9), *p* = 0.010) and lymphocyte count was lower in the SVGS group (2.5 (2.1–3) vs. 2 (1.4–2.8), *p* = 0.034). CRP levels were significantly higher in the SVGS group (2.88 (1.1–6.3) vs. 4 (2.5–7.3), *p* < 0.001).

Markers of inflammation are also summarized in Table 2. NLR, PLR, and GLR values were significantly higher in patients with SVGS (NLR: 1.4 (0.7–3) vs. 2.1 (1.2–3.7), PLR: 92 (69–122) vs. 110 (73–172), GLR: 34.7 (28.2–42.4) vs. 63.7 (49.4–78.1), *p* < 0.001, for all).

The evaluation of conduits used during CABG by CAG is presented in Table 3. There was no significant difference between the two groups in terms of the distal anastomosed coronary vessels (left anterior descending artery, left circumflex artery, right coronary artery) (*p* > 0.05). No statistically significant difference was observed between the groups in terms of the number of LIMAs and SVGs (*p* > 0.05). However, the interval between CABG surgery and CAG was significantly longer in the SVGS group (8.4 ± 1.6 years vs. 7.2 ± 1.8 years, *p* < 0.001).

The efficacy of risk factors for SVGS was assessed by multivariate analysis. Multivariate logistic regression analysis was performed using variables that were associated with disease occurrence in univariate analysis, including DM, blood glucose level, NLR, CRP, PLR, mean time since CABG, and GLR. In this analysis, DM, CRP, mean time since CABG, and GLR were identified as independent predictors of SVGS (Table 4).

**Figure 1:**
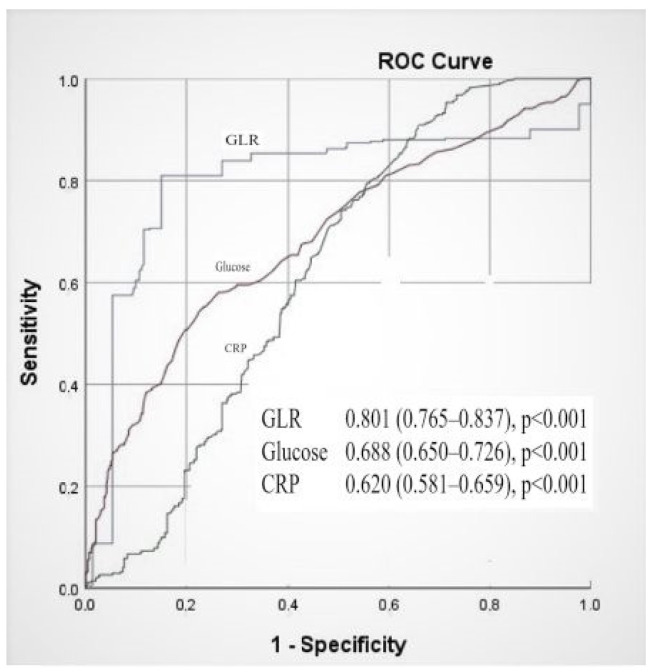
ROC analysis with inflammatory markers in saphenous vein graft stenosis patients.

Receiver operating characteristic (ROC) analysis revealed that a GLR cut-off value > 315.5 predicted SVGS with 75.8% sensitivity and 68.6% specificity (area under the curve (AUC) = 0.801 [95% CI: 0.765–0.837], *p* < 0.001). Similarly, a cut-off value of 470.7 for blood glucose predicted SVGS with 73% sensitivity and 60.1% specificity (AUC = 0.688 [95% CI: 0.650–0.726], *p* < 0.001). For CRP, a cut-off value of 0.95 showed 70.9% sensitivity and 60.7% specificity for predicting SVGS in CABG patients (AUC = 0.620 [95% CI: 0.581–0.659], *p* < 0.001) (Figure 1).

## 4. Discussion

In this study, GLR was found to be an effective biomarker for the detection of SVGS in patients undergoing CABG. In addition, increased prevalence of DM and prolonged mean postoperative time were found to be other important variables affecting the detection of SVGS. Inflammatory markers such as NLR and PLR were also evaluated in the study, and the results show that GLR is a better marker for predicting SVGS compared to NLR and PLR.

In the SVG system, late atherosclerosis becomes an important factor in graft failure. SVGs are known to degenerate over time and show markedly accelerated atherosclerosis even if they remain open. As early as the first year after anastomosis to the arterial system, SVGs show neointimal hyperplasia and accumulation of foamy macrophages. This process progresses to stenotic lesions with enlargement of necrotic cores [19]. Within 5 to 10 years, these neoatherosclerotic plaques become stably established and are often associated with fragility, erosive properties, and luminal thrombus formation [19]. This leads to a high incidence of ischemia-related events, distal embolization, and occlusive pathologies [20].

Inflammation acts as a cascade in all stages of atherosclerosis, playing an important role until the development of complications [21]. The relationship between systemic inflammation and atherosclerosis has been demonstrated in several studies using different markers of inflammation. In particular, there are studies on many biomarkers associated with SVGS in CABG patients. For example, Oksuz et al. [15] found a significant association between LMR and SVGS, while Oguz et al. [22] demonstrated a link between SII and SVGS in the early period. Furthermore, Serhatlioglu et al. [23] observed more SVGS in patients with high PIV, while Yayla et al. [24] reported a significant association between CAR and SVGS.

The GLR is a new inflammation parameter that has been introduced in recent years and studies have been published showing that it can be used as a marker to predict prognosis and disease severity in some disease groups, including cardiovascular diseases [25,26,27,28]. According to the literature, studies examining the relationship between GLR and CAD are still limited, but this newly introduced biomarker stands out as a parameter worthy of more detailed evaluation in future studies. One of the first studies in this field was conducted by Liu et al. [29], who concluded that GLR levels could be a potential predictor for the prognosis of acute myocardial infarction (MI) and could be used for early risk stratification of clinically high-risk populations. Serhatlioglu et al. [30] showed a significant association between GLR and the severity and complexity of coronary artery disease in patients with non-ST segment elevation myocardial infarction (NSTEMI). Considering previous studies on the assessment of inflammation-related progressive disease, this study supports the notion that GLR, a novel marker of inflammation used to predict SVGS, may be a potential predictor of disease severity [31,32,33,34]. Furthermore, in this study, we found that GLR was a more effective biomarker compared to NLR and PLR, which are almost routinely used as inflammation parameters, supporting some other studies [26,28,35]. This result demonstrates the value of GLR as a potential biomarker in predicting the prognosis of cardiovascular diseases and suggests that it may be more widely evaluated in future clinical applications.

GLR is a parameter derived from the ratio of glucose and lymphocyte levels, and it has been suggested that it may be a more powerful parameter than the combination of the predictive values offered by both variables alone. Most of the hematologic or biochemical parameters used to assess the inflammatory cascade are derived from the ratio of similar group variables. However, the derivation of the GLR from the ratio between parameters that have the potential to represent different inflammatory cascades, one metabolic (glucose) and the other hematologic (lymphocyte), suggests the possibility that it may better reflect the activity and severity of the level of inflammation in the host compared to other parameters (NLR, PLR, SII, CAR, etc.). This leads us to believe that the GLR allows a more comprehensive assessment of the inflammatory response and may be a more sensitive indicator for predicting the course of the disease.

The physiopathological mechanisms suggesting that elevated GLR is a better indicator of increased inflammation and stress in the body can be summarized as follows: Lymphocytes participate in inflammation through direct cellular interactions and secreted mediators [36,37]. Decreased cell production and/or redistribution of cells at the tissue level or increased apoptosis may lead to lymphopenia. In the plaque structure, a reduced number of lymphocytes may lead to plaque growth and/or progression, as well as impaired plaque stability [38,39]. Furthermore, lymphocyte count is an important parameter reflecting the nutritional status of the host; thus, decreased lymphocyte count is inversely correlated with the severity of inflammation and may provide information to assess the efficiency of the immune system [40].

Hyperglycemia is considered a metabolic marker associated with chronic subclinical inflammation, defined as “metaflammation” [41]. Chronic subclinical inflammation aggravates hyperglycemia by regulating insulin resistance and leads to a series of diabetic complications. A review has published an association between poorly regulated glucose metabolism and increased levels of chronic inflammatory markers (interleukin-1β, IL-6, and tumor necrosis factor-α) in diabetic patients [42]. High blood glucose and lipid levels cause metabolic disorders and also activate inflammatory cascades that use similar pathways [41,43]. This leads to further metabolic disorders, creating a vicious cycle. Some of the mediators regulating these mechanisms may be as follows. Increased blood glucose levels trigger inflammatory mediators and stress in the intracellular endoplasmic eticulum (ER) [43]. ER stress per se may lead to increased TNF-α expression or perhaps to more general inflammatory responses [44]. Increased intracellular free oxygen radicals lead to greater ER stress, insulin resistance, and greater ROS production, which in turn produce broader inflammatory responses [45,46]. Hyperglycemia also promotes a prethrombotic state, increases inflammation and sympathetic nervous system activity, worsens endothelial function, and causes oxidative stress to release reactive oxygen species, thereby exacerbating coronary artery damage [47,48]. These physiopathologic factors support the idea that the GLR more sensitively reflects increased inflammation and stress states in the body and may therefore be an important predictive parameter.

## 5. Conclusions

It can be suggested that there is a potential relationship between GLR and SVGS, and this ratio can be used in the evaluation of SVGS. In addition, it can be speculated that these and similar inflammatory markers can be used for treatment targeting and/or disease severity assessment once they are introduced into daily clinical practice. However, due to the limited number of patients and indirect assessment of inflammation in this study, studies with larger participation and more patients are needed to increase the accuracy and generalizability of these findings.

### Limitations

The relatively low number of patients participating in this study can be considered as one of the limitations of the study. In addition, its single-center and retrospective design may limit the generalizability of the study results. Since the study did not evaluate mediators and biomarkers such as TGF, NO, and VEGF that affect atherosclerosis and are associated with this disease, the information provided about the potential value of GLR at the tissue level remains limited. Due to the nature of correlation analyses, the findings of this study do not demonstrate causality. In addition, GLR levels were only measured in blood samples taken at the time of admission, and since this study was not a follow-up study, information on how changes in GLR levels evolve over time and the effects of these changes on the disease could not be provided. Some metabolic variables for DM (HbA1c, insulin resistance, etc.) were not included in the study. In addition, the effects of diet and/or medications (e.g., metformin, insulin, or statins) on GLR were not evaluated. In addition, we have no data on the effects of genetic factors on GLR levels. The exclusion criteria for patients with higher risk groups such as “recent myocardial infarction, hematological dissemination, etc.” may have caused a selection bias by not including them in the study. However, the inclusion of only symptomatic and/or ischemia-positive SVGS patients in the study and the exclusion of asymptomatic SVGS patients may have also caused a selection bias.

## Figures and Tables

**Table 1:** Demographic characteristics of the study populations.

	Saphenous Vein Graft Stenosis		
	Non-Existent	Exist	*p* Value
Variables	(n = 437)	(n = 341)	-
Age (years) ^a^	57 (41–67)	57 (48–64)	0.155
Men gender (n, %)	234 (53.4%)	190 (55.7%)	0.546
BMI (kg/m^2^)			
Diabetes mellitus (n, %)	88 (20.1%)	102 (29.9%)	**0.002**
Hypertension (n, %)	182 (41.6%)	152 (44.5%)	0.413
Hypercholesterolemia (n, %)	89 (20.3%)	80 (23.4%)	0.299
Current smoker (n, %)	76 (17%)	68 (19%)	0.363
LVEF (%) ^b^	48.3 ± 8.7	51.4 ± 10.3	0.098
**Postoperative medications**			
Aspirin (n, %)	301 (68.9)	229 (67.2)	0.642
Β-blocker (n, %)	357 (81.7)	275 (80.6)	0.119
Angiotensin–aldosterone antagonists (n, %)	286 (67.8)	242 (70.9)	0.253
Statin (n, %)	258 (59)	224 (65.7)	0.856
Clopidogrel (n, %)	163 (37.3)	115 (33.7)	0.35
Nitrat (n, %)	85 (19.5)	76 (22.3)	0.559

^a^; median-IQR, ^b^; mean ± standard deviation. LVEF: left ventricular ejection fraction, BMI; body mass index.

**Table 2:** Laboratory findings of the study populations.

	Saphenous Vein Graft Stenosis		
	Non-Existent	Existent	*p* Value
Number of Patients	(n = 437)	(n = 341)	
Glucose (mg/dL) ^a^	89 (78–106)	113 (88–160)	**<0.001**
Creatinine (mg/dL) ^b^	1.1 + 4	0.96 + 0.6	0.540
AST (U/L) ^b^	24.3 + 16	24.6 + 17	0.844
ALT (U/L) ^b^	23.6 + 18	26.2 + 16	0.157
Total cholesterol (mg/dL) ^b^	197.2 + 46.4	199.4 + 40	0.502
High-density lipoprotein cholesterol(mg/dL) ^b^	48.7 + 12	48.5 + 12	0.797
Low-density lipoprotein cholesterol (mg/dL) ^b^	117 + 37	120 + 35	0.221
Triglyceride (mg/dL) ^b^	155.4 + 87	159.1 + 87	0.565
Hemoglobin (mg/dL) ^b^	14.0 + 2	14.3 + 2.2	0.452
Platelets (10^3^/µL) ^a^	242 (190–301)	230 (190–293)	0.189
WBC (10^3^/µL) ^a^	6.9 (4.8–10)	7.4 (5.5–10)	0.112
Neutrophil (10^3^/µL) ^a^	3.9 (2–7.4)	4.2 (3–6.9)	**0.010**
Lymphocyte (10^3^/µL) ^a^	2.5 (2.1–3)	2 (1.4–2.8)	**0.034**
NLR ^a^	1.4 (07–3)	2.1 (1.2–3.7)	**<0.001**
PLR ^a^	92 (69–122)	110 (73–172)	**<0.001**
GLR ^a^	34.7 (28.2–42.4)	63.7 (49.4–78.1)	**<0.001**
hs-CRP ^a^	2.88 (1.1–6.3)	4 (2.5–7.3)	**<0.001**

^a^; median-IQR, ^b^: mean ± standard deviation. WBC: white blood cell, NLR: neutrophil/lymphocyte ratio, PLR: platelets/lymphocyte ratio GLR: glucose/lymphocyte ratio, hs-CRP: high-sensitive C-reactive protein.

**Table 3:** Angiographic data.

	Saphenous Vein Graft Stenosis		
	Non-Existent (n = 437)	Existent (n = 341)	*p* Value
Distal Anastomosis Area			
Left anterior descending coronary artery (n, %)	437 (100)	341 (100)	
Left circumflex coronary artery (n, %)	411 (94.2)	315 (92.4)	0.316
Right coronary artery (n, %)	420 (96.1)	311 (92.3)	0.752
**Left internal mammary artery usage (n %)**	435 (99.5)	338 (99.1)	0.891
**Number of saphenous vein grafts (n)**	2.27	2.43	0.02
**Average time after bypass (years)**	8.1 + 1.3	7.02 + 1.4	<0.001

**Table 4:** Univariate and multivariate logistic regression analysis to find independent predictors of saphenous vein graft disease.

	Univariate Analysis			Multivariate Analysis		
	Odds Ratio	95% CI	*p* Value	Odds Ratio	95% CI	*p* Value
**DM**	1.693	1.218–2.352	**0.002**	1.700	1.189–2.431	**0.004**
**Glucose**	1.012	1.009–1.016	**<0.001**			
**Lymphocyte**	1.169	1.009–1.355	**0.037**			
**GLR**	1.018	1.012–1.024	**<0.001**	1.031	1.023–1.040	**<0.001**
**NLR**	1.119	1.053–1.188	**<0.001**			
**PLR**	1.007	1.005–1.010	**<0.001**			
**CRP**	1.037	1.005–1.070	**0.023**	1.035	1.000–1.072	**0.048**
**Average time after bypass (years)**	1.460	1.303–1.635	**<0.001**	1.422	1.249–1.619	**<0.001**

CI: confident interval, DM: diabetes mellitus, GLR: glucose/lymphocyte ratio, NLR: neutrophil/lymphocyte ratio, PLR: platelets/lymphocyte ratio, CRP: high-sensitive C-reactive protein.

## Data Availability

The data that support the findings of this study are available from the corresponding author upon reasonable request.
